# Editorial

**Published:** 1884-04

**Authors:** 


THE annual session of the Medical Association of Alabama will be held in Selma, April 8th, 9th, 10th and 11th.

---

FIFTY-FIRST annual session of the Tennessee State Medical Society will convene at Chattanooga on Tuesday, April 8th, 1884.

---

THE SANITARIAN comes to us now in the form of a monthly octavo of 96 pages. It is a valuable journal and does good service in its special field.

---

DR. LUNSFORD P. YANDELL, senior editor of the Louisville *Medical News*, died suddenly at his home in Louisville, on the morning of March 12th, of angina pectoris.

---

THE ANALECTIC is the name of a new monthly published by G. P. Putnam's Sons, New York, and edited by Walter S. Wells, M. D. It is a handsome journal of 48 pages, and is filled with the latest information on medical matters at home and abroad.

---

Dr. J. F. Goldman, Huntsville, Ala., says: "Was called to see a little girl, eight years old, on January 18, who was troubled with a vaginal discharge of buff color and almost creamy consistency. She complained of pains in her back, hips, and lower abdomen, very much as women do who suffer from leucorrhœa Having had a case of gonorrhœa recently in a girl of similar age, I suspected something of the kind here; but examination proved the suspicion wrong. Further examination and inquiry convinced me that she was of a scrofulous diathesis, with a possible syphilitic origin. Knowing of no alterative so effective in scrofula as Battle & Co.'s IODIA, I ordered half-drachm doses three times per day. In a few days the discharge ceased, and all symptoms passed away."

